# The Physician’s Tears: Experiences and Attitudes of Crying Among Physicians and Medical Interns

**DOI:** 10.1007/s10880-019-09611-9

**Published:** 2019-02-19

**Authors:** Kim M. E. Janssens, Chloë Sweerts, Ad J. J. M. Vingerhoets

**Affiliations:** 1 Tranzo, Scientific Centre for Care and Welfare, Tilburg School of Social and Behavioral Sciences, Tilburg University, Warandelaan 2, 5037 AB Tilburg, The Netherlands; 2 Department of Medical and Clinical Psychology, Tilburg School of Social and Behavioral Sciences, Tilburg University, Tilburg, The Netherlands

**Keywords:** Crying, Tears, Attitudes, Experiences, Physicians, Medical interns

## Abstract

We examined several aspects of the crying experiences of physicians and medical interns, including the most common reasons to cry in the workplace, and their perceptions of and attitudes towards crying in the workplace and in the presence of a patient. A sample of Dutch physicians and medical interns (*N*_physicians_ = 1068 and *N*_medical interns_ = 302 and for the full version *N*_physicians_ = 776 and *N*_medical interns_ = 181) completed an especially designed anonymous online questionnaire about experiences with crying in the workplace, and perceptions of and attitudes towards crying in the workplace and in the presence of patients. Crying is a rather frequent behavior among physicians, in particular when they have to deal with the severe suffering of patients and their relatives. We found a considerable variety in the attitudes and perception of crying in the work setting, although there was also much agreement that crying in the presence of a patient is only appropriate if it is over the condition of the patient. Physicians reported a slightly more positive attitude and a stronger need for more attention to this topic in training and education than medical interns. Crying in the medical setting is a common, though understudied phenomenon. There is a strong need for further research on this topic in order to understand it better and how and when it might interfere with or facilitate with the therapeutic process. We strongly feel that currently the time is ripe for this topic because in particular the physicians expressed a greater need for more attention to this topic in training and the medical interns showed signs of, perhaps unhealthy, suppression of their emotions.

## Introduction

Bringing good or bad news to patients after medical examinations and dealing with the suffering and losses of patients and family members are almost daily tasks of health professionals. Especially when there is intensive contact with a patient, such experiences can affect physicians visibly even to the point that their tears well up. Is such crying by a physician unprofessional or even unethical? Or can physicians also show their human side during such moments? How do these professionals feel about crying in the presence of a patient? Occasionally, the emotions of physicians receive much attention in the media. For example, in 2015, a photograph on the American forum *Reddit.com* of a physician kneeling against a wall outside of the emergency room, mourning about a 19-year-old who just passed away, went viral and evoked many different reactions both from physicians and the general public (Reddit.com, 2015).

In addition, several health professionals and medical students have addressed the topic of crying in the presence of a patient in columns, essays, and online blogs (e.g., Bradley, 2018; Farber, 2018; Gulland, 2014; Johnson, 2017; Lerner, 2008; Sinclair, 2008; Wible, 2015; Winebrenner, 2018). A recurring issue is the felt conflict between professional composure and the maintenance and expression of compassion. The many examples that are provided often suggest that when a physician cries with a patient, they have a common history. A sad event does not seem a sufficient reason to cry, in some way (e.g., because of a similar personal experience, or because for some other reasons) there also must be a kind of connection. In addition, there is agreement that the emotion of the moment should never be about the physician, but rather about the patient and his or her family. Further, the emotion may of course not interfere with the adequate provision of professional help. Moreover, there is an awareness that both health professionals and patients differ enormously in how they perceive and experience a crying health provider. Whereas some patients appreciate an empathic physician, others prefer a physician who can professionally distance him or herself from the suffering of the patients (Wible, 2015). This latter conclusion received support from the results of a Dutch general population survey (Omroep Max, 2015) which nicely reflect the wide variety of opinions of the general public regarding crying physicians. More specifically, 50% disagreed with the statement that crying of physicians in the presence of a patient is appropriate, whereas approximately one-third confirms this statement. That did, however, not imply that crying physicians were regarded as not fit for the job. Around a quarter agreed with that statement, whereas more than 50% perceived crying physicians as still fit for the job. Slightly more than 40% endorsed the statement that a crying physician is a good physician because expressing empathy makes a better physician than distancing oneself from the patient. Remarkable was the striking correspondence in the opinions of men and women.

Among physicians, there is a similar strong disagreement (cf. Blair & Wasson, 2015; Pruthi & Goel, 2014), although there is also some consensus that crying by physicians might be acceptable, but only if it benefits the patient. Whereas some are convinced that showing tears to a patient can help them in processing their sadness and in building a relationship with and supporting the patient, others strongly feel that physicians should never cry in the presence of a patient, not only because it does not help the patient, but also because such a reaction may increase the physician’s risk of burnout (Lerner, 2008). As already said, the emotion of the moment should in all cases be about the patient, not about the doctor, because that would imply an unacceptable role reversal (Lerner, 2008; Sinclair, 2008).

Such mainly anecdotal evidence thus seems to suggest confusion and disagreement among physicians about if and how they should express their emotions while guarding their image as a good professional and their emotional well-being. The increased attention to the emotions of physicians during medical education, as well as the increasing number of health professionals who dare to share their experiences with crying in the workplace (e.g., Bradley, 2018; Farber, 2018; Lerner, 2008; Sinclair, 2008; Wible, 2015; Winebrenner, 2018) shows the relevance of open discourse about crying physicians.

The lack of research addressing this issue thus seems in stark contrast to the obvious interest of the medical field and the general public. In particular, studies exploring this topic more systematically are the exception. Fewer than a handful of empirical studies have examined the experiences with and attitudes towards crying among physicians and/or medical interns. To the best of our knowledge, only Barth, Egger, Hladschik-Kermer, and Kropiunigg (2004) and Wagner, Hexel, Bauer, and Kropiunigg (1997) examined the occurrence of crying among physicians and medical interns in hospitals. Both studies showed that the majority of physicians (respectively 57% and 53.1%) and medical interns (respectively 31% and 37.9%) had ever cried in the workplace. The studies of Angoff (2001), Kukulu and Keser (2006) and Sung et al. (2009) additionally focussed specifically on the crying of medical interns during their internships. Remarkably, these studies among medical interns yielded substantially more variation in the findings (ranging between 31% and 73%) (Angoff, 2001; Barth et al., 2004; Kukulu & Keser, 2006; Sung et al., 2009; Wagner et al., 1997).

Regarding the antecedents of the crying, among the most important triggers for physicians and medical interns are delivering bad news (Barth et al., 2004) and the death of a patient (Barth et al., 2004; Wagner et al., 1997), in particular in the case of children or young adults, or because of the mourning of family (Wagner et al., 1997). Medical interns additionally cry over the patient’s story, but also when experiencing a heavy workload, and over-educational issues, as well as over conflicts with supervisors or colleagues (Angoff, 2001; Barth et al., 2004; Kind, 2013; Kukulu & Keser, 2006; Sung et al., 2009; Wagner et al., 1997).

Earlier research reported rather strong negative attitudes towards crying in the workplace and in the presence of a patient, especially among medical interns (Angoff, 2001; Kukulu & Keser, 2006; Sung et al., 2009). Sung et al. (2009) found that about half of the medical interns regarded it unprofessional to cry in the presence of a patient as a physician or medical intern. They generally considered crying in the work setting more as a sign of weakness and strongly felt that a crying physician is not fit for the job. Not surprisingly, they further expressed the fear that crying in the workplace might have negative effects on their evaluation. This fear is particularly present when they cry out of stress (Angoff, 2001). Turkish research additionally showed that more than half of the medical interns soothe after a crying situation and that they find it pleasant to share their feelings (Kukulu & Keser, 2006). Some final interesting observations were that medical interns were worried that they would lose their compassion to the patient when they could not express their emotions (Angoff, 2001) and that negative reactions, such as being laughed at, after crying as a physician (Barth et al., 2004; Wagner et al., 1997) are exceptional, although there are examples that supervisors made negative comments (Sung et al., 2009).

Crying in the workplace is thus not unusual for physicians and medical interns. However, how common they are, these tears in the workplace nevertheless can make health professionals uncertain about their professionality, emotional stability, and clinical skills (Pruthi & Goel, 2014). In particular, medical interns can feel anxious and shameful when they show their emotions, for example about being unable to do their work well or to behave professionally appropriately (Brenner, 2014). At the beginning of their career, these individuals are exposed to many new challenges, dilemmas, and emotions, which can be very demanding and full of pressure. During their education and internships, the attitudes and guidance of their teachers and supervisors are extremely important (Angoff, 2001). Consequently, the right advice can support medical interns in dealing with their emotions and the situation of a patient, whereas inadequate supervision can easily result in uncertainty and feelings of incapability (Angoff, 2001; Blair & Wasson, 2015).

Recent research suggests that, more generally, also in the work setting, adult crying is seen as acceptable, if the reason for crying is normative (maybe taking oneself as a standard), much similar as feeling grief at funerals or happiness at weddings (cf. Elsbach & Bechky, 2018; Vingerhoets, 2013). In such situations, observers tend to react with understanding and empathy. Crying that is considered as not appropriate, however, may result in substantial negative reactions from observers. In particular in work settings, such crying can easily be labeled as unprofessional and associated with negative outcomes, such as poor performance evaluations and career limitations (Poverny and Picascia, 2009). It seems plausible that similar rather subtle factors also play a significant role in how the crying of physicians and medical interns is evaluated.

Finally, one may wonder about the relationship between crying and burn out, which might be more complex than is often assumed, because crying can be considered in several different ways, such as a sign, a signal, and a symptom (Vingerhoets & Bylsma, 2007). Remarkably, in the literature, there is strong disagreement regarding this issue. Whereas some suggest that crying may predispose an individual to the development of burn-out (e.g., Lerner, 2008), others (e.g., Larson & Yao, 2005) argue that it is the inhibition of emotions and surface acting that might result in negative health consequences.

### The Present Study

The present study examined the occurrence of crying in the workplace and perception of and attitudes towards crying health professionals with a special focus on the differences between physicians and medical students/interns. What is unique in the present study is the differentiation between crying in the workplace (without patients) and crying in the presence of a patient. Therefore, the general research questions of this study were: (1) How often do physicians and medical interns cry (also in the work setting) and what are the common reasons to cry over in the workplace and in the presence of a patient? (2) What are the attitudes of physicians and medical interns towards crying in the workplace and in the presence of a patient and how is such crying perceived? (3) How do the respondents feel about the relationship between crying and burn out? And, finally: (4) To what extent do physicians and medical interns express a need for more attention to this issue in the work setting and in education?

## Method

### Participants

Dutch physicians and medical interns were recruited from February 2015 till March 2015 via social media and through approaching hospitals, medical clinics, and professional associations. The study did not need to be evaluated by the ethics review board review due to the voluntary participation and the anonymity of the online survey.

The data collection consisted of two parts. In total, 1370 Dutch medical professionals (*N* = 1068 physicians, *N* = 302 medical interns) participated in Part I. The mean ages of the physicians and medical interns were, respectively, 42 years (SD = 12.7) and 24 years (SD = 0.3), while the mean years of work experience were 14 years (SD = 11.4) and 1.3 years (SD = 0.9). Table 1 summarizes the demographics of the research sample.

**Table 1:** Demographics of the participants

	Physicians (*N* = 1068)	Medical interns (*N* = 302)	Total (*N* = 1370)
	% (*N*)/*M* (SD)	% (*N*)/*M* (SD)	% (*N*)/*M* (SD)
Gender (men/women (W))	Men: 69.9% (*N* = 746)	Men: 76.8% (*N* = 232)	Men: 71.4% (*N* = 978)
	W: 30.1% (*N* = 322)	W: 23.2% (*N* = 70)	W: 28.6% (*N* = 392)
Age	*M* = 42.0 (SD = 12.7)	*M* = 24.5 (SD = 3.7)	*M* = 38.2 (SD = 13.4)
Years of work experience	*M* = 13.8 (SD = 11.4)	*M* = 1.3 (SD = 0.9)	*M* = 11.0 (SD = 11.3)
Specialism			
General practitioner care	24.2% (*N* = 258)		
Diagnostic specialties	35.5% (*N* = 379)		
Surgical specialties	6.1% (*N* = 65)		
Supportive specialties	7.5% (*N* = 80)		
Gynaecology and paediatrics	17.0% (*N* = 182)		
Other	9.7% (*N* = 104)		
Full- (F)/part-time (P) job	F: 61.0% (*N* = 651)	F: 92.4% (*N* = 279)	F: 67.9% (*N* = 930)
	P: 39.0% (*N* = 417)	P: 7.6% (*N* = 23)	P: 32.1% (*N* = 440)

Part II was completed by 957 respondents (70% of those who started with Part I; 72.6% (*N* = 776) of the physicians and 59.9% (*N* = 181) of the medical interns). Using t tests and Chi square tests, differences on demographic variables between the sample who completed the total questionnaire (*N* = 1370) and the sample who only completed Part I (*N* = 957) were evaluated. Physicians and medical interns who completed the total questionnaire did not significantly differ on gender and working full-time or part-time. However, physicians who completed the total questionnaire had a significantly higher age (*M* = 43.01, SD = 12.85 vs. *M* = 39.44, SD = 11.76) and, consequently, also had more years of work experience (*M* = 14.73, SD = 11.65 vs. *M* = 11.16, SD = 10.32) than physicians who completed only Part I of the questionnaire (age: *t* = 4.307, *p* > .001; years of working experience: *t* = 4.857, *p* > .001). Medical interns who completed the total questionnaire did not differ significantly from medical interns who completed only Part I on age or years of working experience. Table 1 summarizes the background of the study samples.

### Materials and Procedure

Participants completed an anonymous online questionnaire, using the online survey tool Qualtrics (2015). The survey consisted of a brief questionnaire (Part I) and a more extensive Part II. Participants were allowed to stop filling out the questionnaire after Part I (which took about 2 min time), because filling out Part II took about 15 min time. Many of the items (e.g. in Tables 2, 4, 7) were based on the questionnaire applied in a similar study among psychotherapists (‘t Lam, Vingerhoets, & Bylsma, 2018). Part I of the questionnaire contained five questions about demographics and work-related variables (such as years of experience and work specialism). The *attitude towards crying in the presence of patients* was measured with seven statements which were rated on a 7-point Likert scale (1 = strongly disagree, 7 = strongly agree) evaluating the respondents’ attitudes (Cronbach alpha = 0.804; see Table 2). Higher scores represent a more positive attitude. In addition, the presumed relation between crying and burn out and the respondents’ need for more attention to the emotions of physicians in the relationship with patients in education and training was evaluated.

**Table 2:** Attitudes towards crying in the presence of patients—Part I (Cronbach’s alpha = 0.804)

	Total sample (*N* = 1370)		Physicians (*N* = 1068)		Medical interns (*N* = 302)	
	%	*N*	%	*N*	%	*N*
Crying in the presence of a patient is unprofessional	47.4	649	44.7	477	57.0	172
Crying in the presence of a patient is unethical	9.4	129	9.8	105	7.9	24
A physician who cries in the presence of a patient has greater risk to make mistakes and wrong decisions	21.6	296	20.3	217	26.2	79
A physician who cries in the presence of a patients makes himself ridiculous	14.9	204	12.6	135	22.8	69
Crying in the presence of a patient can be a good empathic reaction^a^	59.0	808	52.9	565	80.5	243
Tears in the presence of a patient could be important for the contact between patient and physician^a^	40.9	561	50.5	539	7.3	22
A physician who cries during patient contact is not suitable as physician	13.7	188	6.1	65	40.7	123

^a^Item is reversely coded

Part II of the questionnaire addressed the following topics: (1) personal tendencies to cry and experiences with crying in general; (2) the evaluation and perceived appropriateness of crying at work and related attitudes; (3) the perception of crying physicians, and, finally, (4) participants were asked for what reasons they might cry in the presence of patients.

#### Personal Tendencies to Cry and Experiences with Crying in General

With six questions we evaluated the participants’ frequency of crying in general, in the work place, and in the presence of a patient (see Table 3). The tendency to cry during patient contact was evaluated with three additional questions. We further asked for what reasons they might cry in the presence of patients, using nine 7-point Likert items ranging from 1 (Not likely at all) to 7 (Very likely), with higher scores reflecting greater tendency to cry (see Table 4).

**Table 3:** Percentage of physicians and medical interns crying in the last year and means of how often physicians and medical interns cried during the last year (*N* = 957)

	In general		In the workplace		In the presence of patients	
	%/*N*	*M* (SD)	%/*N*	*M* (SD)	%/*N*	*M* (SD)
Physicians (total sample) (*N* = 776)	87.5% (679)	12.40 (24.65)	48.2% (374)	1.21 (2.27)	25.7% (199)	0.49 (1.20)
General practitioner care (*N* = 199)	87.4% (174)	12.31 (27.66)	45.2% (90)	1.12 (2.11)	35.9% (71)	0.81 (1.76)
Diagnostic specialties (*N* = 265)	90.6% (240)	10.22 (14.01)	46.0% (122)	1.12 (1.87)	16.6% (44)	0.27 (0.72)
Surgical specialties (*N* = 40)	72.5% (29)	7.65 (10.72)	37.5% (15)	0.73 (1.30)	12.5% (5)	0.20 (0.56)
Supportive specialties (*N* = 66)	84.8% (56)	17.80 (32.26)	45.5% (30)	1.11 (2.23)	18.2% (12)	0.36 (0.96)
Gynaecology and paediatrics (*N* = 129)	90.7% (117)	14.28 (31.05)	60.5% (78)	1.47 (2.26)	36.4% (47)	0.61 (1.20)
Other (*N* = 77)	81.8% (63)	14.79 (29.69)	50.6% (39)	1.61 (3.85)	26.0% (20)	0.44 (0.93)
Medical interns (*N* = 181)	93.9% (170)	15.74 (20.39)	47.5% (86)	1.11 (1.76)	12.2% (22)	0.18 (0.58)

**Table 4:** Ratings of situations that could be a reason for physicians and medical interns to cry in the presence of a patient (ratings vary from 1 to 7)

	Physicians	Medical interns	*t*
	*M* (SD)	*M* (SD)	
The touching story of the patient	4.42 (1.76)	4.56 (1.51)	− 1.06
To show the patient how to deal with sadness	1.47 (0.87)	1.38 (0.72)	1.58
To show your involvement to the patient	2.67 (1.70)	2.70 (1.65)	− 0.21
Seeing the patient crying	3.46 (1.76)	4.16 (1.74)	− 4.81***
Seeing the patient suffering	4.28 (1.64)	4.50 (1.52)	− 1.75
Because the treatment of the patient is terminated	3.35 (1.80)	3.59 (1.69)	− 1.63
The story of the patient recalls a personal experience	3.75 (1.80)	4.13 (1.78)	− 2.58**
The patient shows gratitude for the treatment	3.15 (1.65)	2.78 (1.67)	2.70***
The treatment of the patient is more effective than expected	2.92 (1.63)	2.56 (1.57)	2.70***

*p ≤ .05; **p ≤ .01; ***p ≤ .001

Four additional items assessed the feelings after having cried at work, also using 7-point Likert items with 1, respectively, indicating ‘feeling unprofessional, weak, hampered and uncomfortable’ to 7 representing ‘feeling professional, strong, relieved and comfortable.’ Finally, participants were asked if crying in the workplace or in the presence of a patient ever led to any negative consequences (e.g. missing a promotion, salary decrease, or dismissal).

#### Evaluation of and Perceived Appropriateness of Crying in the Work Setting

Fourteen statements addressed additional opinions about *crying in the presence of patients and in the workplace in general* (Cronbach alpha = 0.90; see Table 7). Examples of statements are: ‘A physician needs to have his/her emotions under control in all circumstances’ and ‘When a physician cries during contact with patients, (s)he shows involvement.’ The response format of all statements ranged from 1 to 7, with 1 meaning ‘strongly disagree,’ and 7 meaning ‘strongly agree.’ Higher scores represent a more *negative* attitude towards crying in the presence of patients.

Using eleven 7-point Likert-scale, ranging from 1 ‘inappropriate’ to 7 ‘appropriate,’ the perception of crying in the workplace and in the presence of patients over several reasons (e.g. the bad situation of a patient) was measured (see Table 6a, b). The *appropriateness to cry in the workplace* scale consisted of five items (Cronbach alpha = 0.735). The *appropriateness to cry in the presence of patients’* scale consisted of six items (Cronbach alpha = 0.738). Higher scores on both scales represent a more positive attitude towards crying in the presence of patients.

#### The Evaluation of Crying Physicians

Four statements measured the perception of the *crying of physicians (at work)*, using 7-point Likert-scale, ranging from 1, respectively ‘I think this is inappropriate, unprofessional, ineffective for the work process, and weak’ to 7 respectively ‘I think this is appropriate, professional, effective for the work process, and strong’ (Cronbach alpha = 0.901), with higher scores representing a more positive attitude (see Table 5).

**Table 5:** Evaluations of the crying of physicians (ratings vary from 1 to 7)

	Physicians	Medical interns	*t*
	*M* (SD)	*M* (SD)	
(In)appropriate behaviour	3.74 (1.19)	3.43 (1.14)	3.29***
(Un)professional behaviour	3.66 (1.51)	3.36 (1.11)	3.13**
(In)effective for the work process	3.67 (1.23)	3.64 (1.12)	0.32
Weak/strong behaviour	3.92 (1.12)	3.75 (1.14)	1.83

*p ≤ .05; **p ≤ .01; ***p ≤ .001

Part II additionally contained questions about the work atmosphere, the frequency of crying by male and female colleagues, the expected attitude of patients about crying physicians, the frequency of crying by patients and the attitude towards crying patients. In addition, participants had the opportunity to describe their last crying situation in five open questions. Because these questions do not address the here reported research questions, they are not discussed in the present paper.

### Statistics

Data were analyzed with SPSS 22.0 for Windows (IBM Corp., 2013). Descriptive statistics were computed, using percentages of respondents who agreed or disagreed with the dichotomous questions and mean scores on the 7-point Likert scale questions. For the questions with a 7-point Likert scale, a score of 5 or higher is considered as an agreement, and therefore, as positive, and a score of 3 or lower is regarded as a disagreement and therefore as negative, while a score of 4 is scored as neutral. In addition, scale scores were computed. If the Cronbach alphas were sufficiently high (≥ 0.70), we applied two sample t tests with correction of the degrees of freedom in case of unequal variances to compare the total scale scores of both groups.

## Results

### Part I

Table 2 represents the items that the participants answered in the first part of the questionnaire (M scale = 22.37, SD = 7.36). The data reveal that the respondents tend to be relatively positive towards crying in the presence of a patient, but there is substantial variation. The medical interns reached a statistically significant more negative score than the physicians (respectively *M* = 25.46; SD = 4.79 and *M* = 21.50; SD = 7.72; *t* (786.243) = − 10.909, *p* ≤ .001).

Considerably fewer medical interns (36.1%, *N* = 109) than physicians (87.3%, *N* = 932) indicated the need for more attention to this topic during training and education.

#### Burn Out

Whereas half of the interns (50.3%, *N* = 152) endorsed the statement that crying in the presence of a patient might increase the risk of burn out, only just more than a quart of the physicians (26.3%, *N* = 281) agreed with it, *t* (524,259) = − 8.336, *p* ≤ .001.

### Part II

We first analyzed how common it is for physicians and medical interns to cry (in general, in the workplace, and in the presence of patients), the crying inducing potential of different situations, and what are common reasons to cry in the presence of a patient. Further, we examined what effects their crying had on how they felt after the crying episode. Finally, we addressed how free the participants felt to cry in the presence of a patient and if they had experienced any negative consequences of their crying.

#### Frequency, Antecedents, and Effects of Crying

As many as 88.7% of the participants (*N* = 849) reportedly had cried at least once in the last year (physicians: 87.5%, *N* = 679; medical interns: 93.9%, *N* = 170). Moreover, 48.2% of the participating physicians (*N* = 374) had cried at least once in the workplace in the last year. A further more detailed analysis showed that crying was most often reported in gynecology and pediatrics (50.6%, *N* = 78). In surgical specialties, the lowest percentage of crying physicians was found (37.5%, *N* = 15). Of the medical interns, a comparable percentage of 47.5% (*N* = 86) of the participants had cried at least once in the workplace in the last year. Specifically, in the presence of patients, 25.7% of the physicians (*N* = 199) and 12.2% of the medical interns (*N* = 22) had shed tears at least once in the last year. In the general practitioner care (35.9%, *N* = 71) and, again, in gynecology and pediatrics (36.4%, *N* = 47) crying in the presence of patients was reported most often. For surgical specialties, there was again the lowest percentage of physicians crying (12.5%, *N* = 5). See Table 3 for a summary of the frequencies of crying.

Participants were also asked over which specific situations they may cry in the presence of a patient. Table 4 displays the results. The touching story of the patient, seeing the patient crying, and seeing the patient suffering were rated as the most likely reasons. Least likely were situations such as ‘to show the patient how to deal with sadness,’ ‘to show your involvement to the patient,’ ‘the patient shows gratitude for the treatment,’ and ‘the treatment of the patient is more successful than expected.’

None of the medical interns reportedly has had negative consequences such as a missed promotion, salary decrease or dismissal after crying in the work setting or during patient contact. Of the physicians, 1% (*N* = 8) did experience such negative consequences after crying in the workplace or during patient contact.

A considerably lower percentage of medical interns than of the physicians felt free to cry in the work setting (6.1% vs. 30.9%) and in the presence of a patient (3.4% vs. 13.2%). The medical interns additionally reported more suppression of one’s emotions at work (34.7% vs. 21.2%) and not to feel free to cry at work or in the presence of patients (*M* scale = 3.55, SD = 1.25).

#### The Perception of Crying in the Workplace and in the Presence of Patients

The crying of physicians is rated as slightly inappropriate and unprofessional, ineffective for the work process, and weak. The ratings of (in)appropriateness and (un)professionality of medical interns were significantly more negative than those of physicians (see Table 5).

Whereas common antecedents of crying (private circumstances, conflict in the work setting, work overload, negative feedback, the bad situation of the patient) are all rated as reasonably suitable to cry over in the work setting, in the presence of a patient, only the bad situation of the patient is regarded as appropriate, whereas all others reasons are seen as most inappropriate (see Table 6a, b).

**Table 6:** Rated appropriateness of reasons to cry at work without the presence of patients (a) and in the presence of patients (b) (ratings vary from 1 to 7)

	Physicians	Medical interns	*t*
	*M* (SD)	*M* (SD)	
(a) Without the presence of patients			
Private circumstances	4.30 (1.66)	4.14 (1.74)	1.12
Conflict in the work setting	4.09 (1.44)	3.93 (1.37)	1.38
Work overload	3.52 (1.44)	3.35 (1.43)	1.36
Negative feedback	3.75 (1.46)	3.51 (1.49)	1.92
Bad situation of the patient	4.46 (1.47)	4.52 (1.31)	− 0.52
(b) Presence of patients			
Private circumstances	1.57 (1.03)	1.69 (1.20)	− 1.15
Conflict in the work setting	1.47 (0.85)	1.42 (0.74)	0.66
Work overload	1.55 (0.89)	1.48 (0.85)	1.07
Negative feedback	1.48 (0.85)	1.46 (0.75)	0.32
Bad situation of the patient	4.29 (1.55)	4.15 (1.47)	1.08

The attitude measure in Part II once more confirmed the picture that medical interns are slightly, but significantly more negative about crying during patient contact (see Table 7 for all items), with scale scores of, respectively, 48.39 (SD = 14.18) and 53.07 (SD = 13.51) for physicians and medical interns, *t* (955) = 4.033, *p* ≤ .001.

**Table 7:** Attitudes towards crying in the presence of patients—Part II (Cronbach’s alpha = 0.900)

	Corrected item–total correlation	Total sample (*N* = 957)		Physicians (*N* = 776)		Medical interns (*N* = 181)	
		%	*N*	%	*N*	%	*N*
When a physician cries in the workplace, (s)he cannot handle the job	0.49	15.6	149	14.9	33	18.2	33
A physician has to take his/her emotions under control in all circumstances	0.41	29.5	282	30.4	46	25.4	46
A good physician should always take distance from the patient and his/her problems	0.45	31.7	303	31.4	59	32.6	59
A physician may also sometimes cry during a patient contact^a^	0.78	49.9	478	53.6	62	34.3	62
When a physician cries during a patient contact, this prevents adequate medical treatment	0.67	24.5	234	22.3	61	33.7	61
During a patient contact, professional distance is more important than empathy	0.38	8.4	80	7.0	26	14.4	26
Tears of a physician may be visible to the patient^a^	0.77	56.5	541	60.2	74	40.9	74
The tears of a physician are a useful tool during a patient contact^a^	0.51	15.3	146	16.4	19	10.5	19
I believe that the tears of a physician (during a patient contact) are experienced as very stressful by the patient	0.55	27.7	265	27.2	54	29.8	54
In contact with patients you should be as authentic as possible and be yourself, even if this means that you sometimes have to leave tears^a^	0.63	56.6	542	58.8	86	47.5	86
When a physician cries during a patient contact, (s)he shows involvement^a^	0.65	67.8	649	67.7	124	68.5	124
When a physician cries during a patient contact, (s)he shows inappropriate involvement and sympathy	0.67	24.1	231	22.0	60	33.1	60
Tears of a physician during a patient contact may be important for the contact between patient and physician^a^	0.68	47.2	452	51.0	56	30.9	56
A physician who cries during a patient contact is too emotional to be able to lead patient contact properly	0.66	31.9	305	29.8	74	40.9	74

^a^Item is reversely coded

## Discussion

Physicians and medical interns have in their daily work to deal with the emotions of their patients but also with their own emotions. The present study is the first to address several aspects of the crying of physicians in the medical setting more systematically. We not only focused on the frequency and common antecedents of crying in the medical setting, but we also assessed attitudes and perceptions and collected information about the experiences with this behavior. Finally, we evaluated the presumed relationship with burn out.

The most important results of this study can be summarized as follows: (1) physicians and medical interns cry relatively often in the workplace, but considerably less in the presence of a patient; (2) In particular medical interns do not feel free to cry and report a relatively strong tendency to suppress their emotions; (3) there is substantial agreement that crying in the presence of a patient is only acceptable when it is about the situation of the patient; (4) after having cried, health professionals tend to feel unprofessional, weak, hampered, and uncomfortable; (5) the attitude towards crying in the presence of a patient is moderately positive, on average, but that holds more for physicians than for medical interns; and (6) there is also substantial disagreement concerning the question of whether a greater crying proneness may predispose to burn out, with again a remarkable difference between physicians and medical interns, who consider this crying as a greater risk.

Whereas the vast majority of the participating physicians feel that the emotions of a physician in the relationship with patients should receive more attention in education and training, medical interns do not express such a need for more attention to this subject. However, in the studies of Angoff (2001) and Sung et al. (2009), medical interns noted that they like to share their experiences with fellow students, physicians, and supervisors and engage in discussions with them. Whereas in a previous Turkish study (Kukulu & Keser, 2006), medical interns reportedly would welcome more guidance on the topic of crying in the workplace to help them to better deal with emotions in the workplace, the current Dutch study shows an entirely different picture. Perhaps, in the Netherlands, medical interns feel that there is currently sufficiently attention in the medical curriculum to this topic than in earlier years and maybe also in other countries. However, at the same time, the data seem to suggest that the interns struggle more with the expression of emotions, as evidenced by their more negative attitudes and their stronger conviction that crying in the presence of a patient may be a greater risk to develop a future burn out.

Another possible explanation is that these future physicians attach less importance to this topic and/or feel more uncertain about the medical–technical aspects of their profession. According to earlier Australian research, approximately 25% of the physicians would like to receive psychological support to better understand and deal with their emotions in relation to the patient, especially when it concerns crying (Wagner et al., 1997). In the present study, we found that twice as many physicians reported this need. It is not easy to give a plausible explanation for this difference.

Compared to earlier research (Angoff, 2001; Barth et al., 2004; Kukulu & Keser, 2006; Sung et al., 2009; Wagner et al., 1997), we see some striking correspondences in results on the frequency of crying among physicians. In all studies, approximately half of the participants reportedly ever cried in their work setting. Remarkably, the results found in medical interns show considerably more variation (ranging between 31% and 73%) (Angoff, 2001; Barth et al., 2004; Kukulu & Keser, 2006; Sung et al., 2009; Wagner et al., 1997), with our finding in between, very comparable to the physicians. Crying in front of a patient occurred less often, in particular among medical interns, whereas the crying in both groups was comparable when it concerned crying in general and crying in the work setting. This also may be an indication that the medical interns spend considerable, unhealthy effort to control their emotions, when in the presence of patients. The differentiation between sub-disciplines also reveals some remarkable differences, but given the low sample sizes, one cannot attach too much value to these findings.

The antecedents generally follow the expected pattern with the touching story of the patient and witnessing his or her suffering as strong elicitors of tears. Interestingly, positive events such as when the patient expresses gratitude or when the treatment yields better results than expected are stronger triggers for the physicians than for the interns, whereas seeing the patient crying and when there is an appeal to personal experiences the medical interns are more likely moved to tears. This finding nicely reflects the development of positive crying which becomes more prominent at an older age (cf. Rottenberg and Vingerhoets, 2012).

Physicians and medical interns themselves have in general slightly negative attitudes about a crying physician. Both groups tend to feel that crying in the workplace is not appropriate, unprofessional, ineffective for the work process and a sign of weakness. Medical interns were more negative in their evaluation regarding the (in)appropriateness and (un)professionality of this behavior.

There was also a strong agreement between both groups regarding what was appropriate to cry over in the work setting and in front of a patient. Our findings reveal that physicians and medical interns generally feel that it is appropriate to cry over the bad situation of a patient, both in the presence of patients and in the workplace without patients. In contrast, other reasons, such as private circumstances or conflicts at work are deemed not appropriate to cry about in the presence of a patient. Both physicians and medical interns report that they probably would cry mostly about the (bad) situations of patients. Empathy and compassion, as evidenced by tears, according to a subgroup of the respondents, can certainly facilitate the physician–patient relationship and may contribute to a healing climate.

The findings generally support the view that how a specific crying episode is considered depends on rather subtle factors that play a role. For example, it is clear that the particular cause of the tears and whether or not it occurs in the presence of a patient all seem factors that co-determine how the tears of health professionals are perceived. Further, if the crying is so intense that it interferes with adequately professional acting, a clear limit has been reached. In that sense, the current results illustrate how each crying episode is evaluated on the basis of what Elsbach and Bechky (2018) refer to as scripts. Much similar as in other work settings, the feeling that a “script” is violated triggers much more negative reactions than when everything occurs according to the implicit scripts.

Interestingly, although a significant proportion of physicians seems to be convinced that crying (in the presence of a patient) may increase the risk of burnout (see also Lerner, 2008; Kukulu & Keser, 2006), this conviction is in contrast to the evidence that it is, in particular, the inhibition of emotions, including the suppression of one’s tears and surface acting that may negatively influence one’s well-being (Consedine, Magai, & Bonanno, 2002; Larson & Yao, 2005). Although the medical interns do not feel a strong need for more education on this topic, one may wonder whether additional education on specific this issue is badly needed in this group.

The need for more guidance and education regarding how to deal with one’s emotions as a health professional is considerably stronger among older and more experienced physicians, whereas the interns do not seem to have such a need for more attention in common with them. Therefore, training programs and organizing special meetings for medical professionals on this theme should be focused in particular on the more experienced physicians.

Given the broad variety of opinions, both among physicians (this study) and patients (Omroep Max, 2015), there might be a serious risk that there is not an optimal match between both parties, which might occasionally have negative consequences for the physician–patient relationship and, consequently, the healing process of the patient. That is why the tears of a health professional ask for explicit attention, once it has occurred. If a health professional becomes tearful or cries, (s)he should not leave that unaddressed but discuss it with the patient, to make it more acceptable and understandable for the patient and to avoid that the patient may consider the physician as not professional or, even worse, feel burdened with the physician’s emotions, resulting in a kind of role reversal (e.g., ‘t Lam et al., 2018).

When evaluating the present contribution, readers need to be aware of some limitations. First, data collection was done with an anonymous questionnaire. For that reason, we do not know precisely who completed the survey and who started but did not fully complete it. We thus do not know how representative the sample is for the Dutch population of physicians and medical interns. It seems plausible that this topic especially attracts the attention of physicians and medical interns who in some way have either strongly positive or negative opinions. We also do not know to what extent the answers were biased due to forgetting or factors such as social desirability. These limitations stress the need for further research with different methods to obtain more insight into how, in the medical setting, health professionals deal with their emotions and how others—both superiors, colleagues, and patients—may perceive these tears. In future studies, several aspects of the specific setting should be examined, and the use of video recordings of actual emotional situations, with manipulations of some factors, might be helpful to learn more about the subtle factors that seem to play a role.

In conclusion, the present study supports earlier findings of the regular occurrence of crying in the workplace among physicians and medical interns. It further shows for the first time that medical professionals also cry in the presence of patients and that such crying has a major impact on how they feel themselves. A further finding was that medical interns seem to feel more negative about crying in the presence of a patient and consequently spend more unhealthy effort to control to it and feel worse than physicians, once it does occur. Proper training and education on this topic are essential to foster confidence in health professionals and make them feel more comfortable with dealing with their emotions, even if this is associated with tears.
